# Age related inverse dose relation of sedatives and analgesics in the intensive care unit

**DOI:** 10.1371/journal.pone.0185212

**Published:** 2017-09-28

**Authors:** Amartya Mukhopadhyay, Bee Choo Tai, Deepa Remani, Jason Phua, Matthew Edward Cove, Yanika Kowitlawakul

**Affiliations:** 1 Division of Respiratory and Critical Care Medicine, University Medicine Cluster, National University Health System, Singapore, Singapore; 2 Department of Medicine, Yong Loo Lin School of Medicine, National University of Singapore, Singapore, Singapore; 3 Saw Swee Hock School of Public Health, National University of Singapore and National University Health System, Singapore, Singapore; 4 Alice Lee Centre for Nursing Studies, National University of Singapore and National University Health System, Singapore, Singapore; Azienda Ospedaliero Universitaria Careggi, ITALY

## Abstract

Sedative and analgesic practices in intensive care units (ICUs) are frequently based on anesthesia regimes but do not take account of the important patient related factors. Pharmacologic properties of sedatives and analgesics change when used as continuous infusions in ICU compared to bolus or short-term infusions during anesthesia. In a prospective observational cohort study, we investigated the association between patient related factors and sedatives/analgesics doses in patients on mechanical ventilation (MV) and their association with cessation of sedation/analgesia. We included patients expected to receive MV for at least 24 hours and excluded those with difficulty in assessing the depth of sedation. We collected data for the first 72 hours or until extubation, whichever occurred first. Multivariate analysis of variance, multivariate regression as well as logistic regression were used. The final cohort (N = 576) was predominantly male (64%) with mean (SD) age 61.7 (15.6) years, weight 63.4 (18.2) Kg, Acute Physiology and Chronic Health Evaluation II score 28.2 (8) and 30% hospital mortality. Increasing age was associated with reduced propofol and fentanyl doses requirements, adjusted to the weight (p<0.001). Factors associated with higher propofol and fentanyl doses were vasopressor use (Relative mean difference (RMD) propofol 1.56 (95% confidence interval (CI) 1.28–1.90); fentanyl 1.48 (1.25–1.76) and central venous line placement (CVL, RMD propofol 1.64 (1.15–2.33); fentanyl 1.41 (1.03–1.91). Male gender was also associated with higher propofol dose (RMD 1.27 (1.06–1.49). Sedation cessation was less likely to occur in restrained patients (Odds Ratio, OR 0.48 (CI 0.30–0.78) or those receiving higher sedative/analgesic doses (OR propofol 0.98 (CI 0.97–0.99); fentanyl 0.99 (CI 0.98–0.997), independent of depth of sedation. In conclusion, increasing age is associated with the use of lower doses of sedative/analgesic in ICU, whereas CVL and vasopressor use were associated with higher doses.

## Introduction

The majority of critically ill patients on mechanical ventilation (MV) require medications to reduce pain, agitation and anxiety in the intensive care unit (ICU). Analgesics and sedatives are routinely used for this purpose [1]. Commonly used sedatives are propofol, dexmedetomidine and benzodiazepines [1, 2]. However, benzodiazepines are associated with prolonged duration of MV, increased ICU length of stay (LOS) and development of delirium [3, 4]; therefore benzodiazepine based sedation is not recommended [5]. Duration of MV and LOS in ICU are also adversely affected by excessive sedation [6–8] and the current practice guidelines recommend maintaining “light” sedation [5], as measured by the Richmond Agitation-Sedation Scale (RASS) or Sedation-Agitation Scale (SAS). In addition, when patients are unable to report pain, the Behavioral Pain Scale (BPS) or Critical Care Pain Observation Tool (CPOT) is recommended to ensure analgesic dosage is carefully titrated [9–11]. However, despite these recommendations sedation and pain assessments are not performed routinely in many patients. One survey reported that such assessments were absent in more than 50% of patients [1] and published guidelines indicate that only 60% of ICUs in the United States have adopted the use of a pain-agitation-delirium (PAD) protocol [5]; reported barriers include lack of physician ordering and proper nursing support [12].

Patient related factors like age, gender and weight are important parameters that determine the effective dose of sedatives and analgesics used during anesthesia. As such the target controlled infusion system for propofol, routinely used in the operating room takes into account both age and gender [13]. Higher peak concentrations of propofol are observed in the elderly patients and due to its lipophilic properties, volume of distribution is larger in female patients [14, 15]. However, the current PAD guidelines for adult ICU patients [5] do not recommend dose adjustments according to patient age and gender. In contrast, the 2010 German sedation and analgesia ICU guidelines recommend lower doses for elderly patients, but still fall short of suggesting different dosing based on gender [16]. Therefore, current sedation and analgesia practices in ICU are less sophisticated than those used in anesthesia.

Simple extrapolation of operating room practices to the ICU will not account for altered pharmacokinetic properties when drugs are used in continuous infusions lasting many hours to days. For example, doubling of the 50% effect-site decrement time is observed in elderly patients when propofol infusions are given for 4 hours or more compared to 1 hour [17]. In addition, while the peak effect site concentration of fentanyl is reached with 3.5 minutes when it is delivered as a bolus, steady state concentration takes several hours when infusions are used potentially resulting in higher doses than necessary, and a slower recovery [18]. In the current study, we aimed to examine the effects of patient related factors (e.g. age, gender and weight) and procedures (e.g. central venous line (CVL), restrainers and dialysis) on the doses of common sedatives and analgesics in an ICU already using a sedation and analgesia protocol, and determine if the doses used have any effect on cessation of these medications.

## Materials and methods

### Study cohort and set up

This was a prospective observational cohort study conducted in a 12-bed medical ICU (MICU) between August 2012 and December 2014. The study was approved by the domain specific ethics review board of the National Healthcare Group (Ref: 2012/00818). Requirement of the consent was waived by the ethics committee since the study was analysis of the existing database and did not interfere with patients’ management in anyway. All adult patients (≥18 years) expected to receive invasive MV for at least 24 hours were included in the study. We excluded patients who were admitted following drug overdose, seizure, cardiac arrest, cerebrovascular accident or other neurological diseases resulting in inaccurate assessment of consciousness. Patients transferred from other units and those with long term tracheostomies were also excluded. The MICU is a level 1 closed unit managed by two teams, each consisting of one consultant, one fellow, 3 to 4 residents and 1 to 2 advanced practice nurses. Out of hour services are provided by one fellow and two residents with off-site consultant coverage. Staff nurse to bed ratio varies from 1:1 to 1:2 depending on patient acuity.

### Sedation and analgesia protocol

The sedation and analgesia protocol used in MICU is based on the Society of Critical Care Medicine and German Society of Anesthesiology and Intensive Care Medicine guidelines [16, 19]. All intubated patients are started on analgesia and sedation unless contraindicated. Propofol is the first line sedative, administered via a large bore cannula or CVL at 5–80 μg/Kg/min, titrated every 5 minutes to target a RASS score of 0 (alert and calm) to -2 (light sedation) and recorded every 4 hours. Additional bolus doses are applied according to the bedside nurse’s discretion, or as directed by physicians (e.g. for procedures). When patients are over-sedated (RASS < -2, except during neuromuscular blockade), sedative is stopped until the patient reaches the targeted RASS score and then restarted at half of the previous dose. Periodic monitoring of serum triglycerides is performed in patients receiving more than 200 mg/hour of propofol and cessation considered if triglyceride levels exceed 4.5 mmol/L. Fentanyl is the recommended first line analgesic and is administered intravenously via large bore cannula or CVL at 0.7–10 μg/Kg/hour titrated according to the nurse’s assessment of pain. Bolus doses (0.35–1.5 μg/Kg) of fentanyl are given at the discretion of the bedside nurse and physician. Dexmedetomidine is considered as a second line sedative, used either as an add-on or where delirium and agitation are preventing weaning of other sedatives. Dexmedetomidine is started at 0.15 μg/Kg/hour titrated to a target RASS score; a loading dose of 1μg/Kg over 10min is used in patients not already receiving a sedative. Non-pharmacological measures, talking to the patient and reassurance by bedside nurses, are encouraged. Benzodiazepines are not recommended [3, 5, 20] unless medically indicated for seizure control or as a bolus for procedures. Deep sedation is defined as RASS ranging from -5 to -4.

### Cessation of sedation

Intravenous sedation is routinely stopped every morning by the bedside nurse, unless the patient is using high fraction of inspired oxygen ≥0.8, on neuromuscular blockade, being nursed in the prone position, or has seizures and assessed for spontaneous breathing trial (SBT). Intravenous analgesia is also stopped, unless required for significant pain. If it is not possible to extubate the patient, analgesics with or without sedatives are restarted at half the previous dose and adjusted accordingly to achieve the goals of sedation and analgesia. For the purpose of the study, we defined cessation of sedation as complete stoppage of the medications without any restart.

### Data collection

All data from bedside monitors and devices (e.g. mechanical ventilators, dialysis machines etc.) is recorded into a clinical information system (IntelliSpace Critical Care and Anesthesia, ICCA, Philips Healthcare) and validated by the bedside nurse in real time. Additional data, such as patients’ height, weight, procedures, use of MV, vasopressor, dialysis, restrainer, mobilization, Acute Physiology and Chronic Health Evaluation (APACHE) II and drug infusion rates are entered by the bedside nurse and validated by a second nurse. Medications are dispensed by an electronic dispensing system (Omnicell Pharmacy Dispensing Systems, Omnicell Inc, CA, USA) which monitors individual patient’s name, dose, date/time of dispensing and log in details of the nurse obtaining the medication. We obtained details of the medications from both the ICCA and Omnicell system. Total daily doses of propofol, fentanyl and dexmedetomidine were calculated by adding all the medications used during intravenous infusions and any additional bolus dose(s) used. Sedatives and analgesics used during the first 72 hours of MV were collected for the purpose of this study. Patients were followed up till they were discharged from the hospital or died. We extracted the demographics, diagnosis, LOS and outcomes from the computerized database used in the hospital (Computerized Patient Support System, CPSS, Singapore [21]).

### Statistical analysis

Continuous variables were reported as mean (standard deviation, SD) or median (interquartile range, IQR) where appropriate. Differences in demographic and clinical characteristics between age quartiles were compared using the χ^2^ test for categorical variables. For continuous variables, the ANOVA test was used if the variables were normally distributed; otherwise, the Kruskal-Wallis test was implemented. As the weight-adjusted total dose of propofol and fentanyl were both skewed, the natural logarithmic transformation was applied to normalize the data. Multivariate analysis of variance (MANOVA) was applied to take into account correlated outcomes. The effect of risk factors on the respective total doses/Kg were obtained from multivariate regression models and quantified in terms of relative mean difference (RMD) and its 95% confidence interval (CI). Logistic regression was used to identify factors associated with cessation of sedation and analgesia. The effect of specific risk factors for sedation cessation was quantified based on the odds ratio (OR) and its associated 95% CI. In both MANOVA and logistic regression, risk factors that were significant at the 0.05 level in the univariate analyses were considered for further inclusion in the multivariate analysis. All statistical analyses were generated using STATA v14 (STATA Corp., College Station, TX), assuming a two-sided test at the conventional 5% level of significance.

## Results

**Fig 1:**
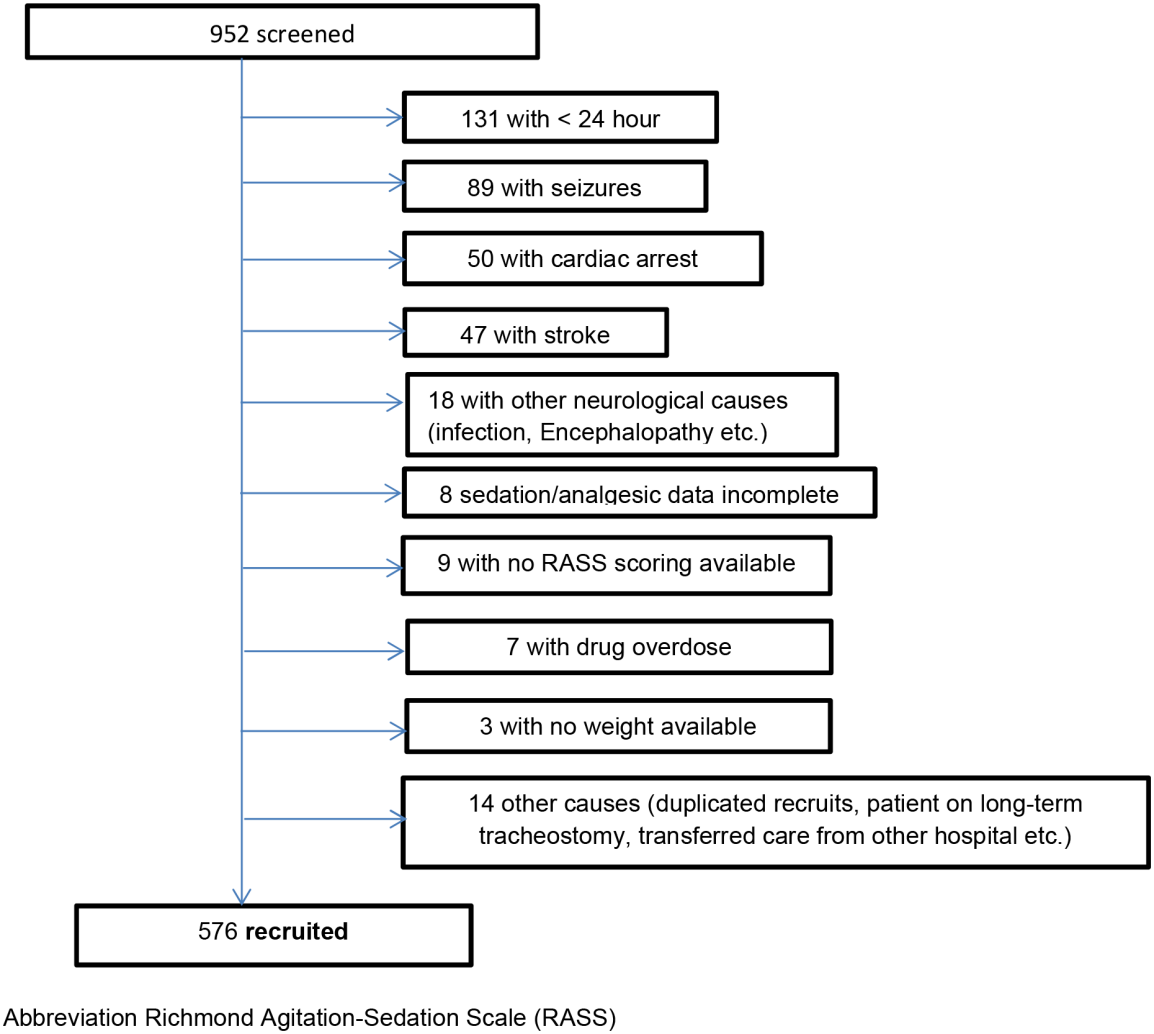
Flowchart showing patient inclusion in the study.

We screened 952 patients, 376 were excluded resulting in a final dataset of 576 patients (Fig 1). The cohort was predominantly male (64%) and ethnically Chinese (58.7%) with mean (±SD) age 61.7 (±15.6, range 18–97) years, weight 63.4 (±18.2) Kg, APACHE II score 28.2 (±8.1) and hospital mortality 30%. Patients were divided into age quartiles for analysis. Table 1 presents patient demographics, outcomes, LOS and ICU procedures for each quartile. There were significant differences between the age quartiles for gender, race, weight, APACHE II score, use of dialysis, vasopressor, transport and arterial lines. Deep sedation was required more frequently in younger age groups. Table 2 presents details of daily medication doses adjusted to weight. Significant differences were noted between daily total dose of propofol and fentanyl in the groups with older patients receiving lower doses of both medications.

**Table 1:** Demographics, procedures and outcomes.

Patient characteristics		Age (years)				p
	All	≤54	55–64	65–74	≥75	
	(n = 576)	(n = 157)	(n = 153)	(n = 151)	(n = 115)	
**Demographics**						
Male (%)	368(63.8)	114(72.6)	99(64.7)	93(61.6)	62(53.9)	0.015
Race (%)						<0.001
Chinese	338(58.7)	62(39.5)	87(56.9)	104(68.9)	85(73.9)	
Malay	135(23.4)	44(28.0)	43(28.1)	27(17.9)	21(18.3)	
Indian	57(9.9)	28(17.8)	13(8.5)	10(6.6)	6(5.2)	
Others	46(8.0)	23(14.7)	10(6.5)	10(6.6)	3(2.6)	
Mean weight, Kg, (SD)	63.4(18.2)	67.8(22.0)	65.7(19.4)	61.4(14.4)	57.1(12.2)	<0.001
Mean APACHE II, (SD)	28.2(8.1)	25.4(8.1)	27.8(8.6)	30.3(7.3)	29.7(7.3)	<0.001
**Outcomes**						
ICU mortality (%)	120(20.8)	31(19.9)	33(21.6)	33(21.8)	23(20)	0.96
Hospital mortality (%)	171(29.6)	37(23.6)	42(27.4)	50(33.1)	42(36.5)	0.08
Median ICU LOS, days, (IQR)	6(4–9)	6(3–10)	6(4–9)	6(4–9)	6(4–10)	0.74
Median hospital LOS, days, (IQR)	17(9–31)	17(8–29)	19(10–36)	16(9–26)	16(10–30)	0.42
**ICU Procedures**						
Median duration of Mechanical ventilation, hours, (IQR)	78(46–142)	81(50–129)	95(55–158)	64(42–132)	78(46–142)	0.069
Use of Restrainer (%)	319(55.4)	85(54.1)	91(59.5)	80(52.9)	63(54.7)	0.68
Dialysis (%)	154(26.7)	37(23.6)	56(36.6)	39(25.8)	22(19.1)	0.008
Vasopressor (%)	415(72.0)	96(61.2)	116(75.8)	117(77.5)	86(74.8)	0.005
Transport (%)	55(9.5)	10(6.4)	18(11.8)	21(13.9)	6(5.2)	0.036
Mobilization (%)	51(8.9)	12(7.6)	16(10.5)	17(11.3)	6(5.2)	0.291
Central Venous Line (%)	537(93.2)	143(91.1)	147(96.1)	142(94.0)	105(91.3)	0.267
Arterial Line (%)	552(95.8)	145(92.4)	148(96.7)	149(98.7)	110(95.7)	0.043
NG/OG Tube (%)	562(97.6)	151(96.2)	150(98.0)	150(99.3)	111(96.5)	0.267
Deep Sedation Required (%)						
Day 1	175(30.2)	68(38.9)	39(22.3)	44(25.1)	24(13.7)	0.002
Day2*	146(26.3)	53(36.3)	37(25.4)	39(26.7)	17(11.6)	0.006
Day3**	86(21.4)	33(38.4)	21(24.4)	26(30.2)	6(7.0)	0.002

Abbreviations SD standard deviation, APACHE Acute Physiology and Chronic Health Evaluation, ICU intensive care unit, LOS length of stay, IQR interquartile range, NG/OG naso/oro-gastric.

*N = 555555.

**N = 401.

**Table 2:** Details of medications.

	Age (years)				
	≤54	55–64	65–74	≥75	p
**Medications**	**Day 1**				
	**n = 157**	**n = 153**	**n = 151**	**n = 115**	
Mean Propofol (mg/Kg), (SD)	62.84(55.86)	46.45(51.27)	40.09(38.08)	26.88(32.44)	<0.001
Mean Fentanyl (mcg/Kg), (SD)	63.82(52.99)	48.83(45.96)	45.61(36.32)	32.52(26.99)	<0.001
Mean Dexmedetomidine (mcg/Kg), (SD)	0.05(0.49)	0.04(0.38)	0.05(0.63)	0.04(0.27)	<0.001
	**Day 2**				
	**n = 151**	**n = 148**	**n = 149**	**n = 107**	
Mean Propofol (mg/Kg), (SD)	28.38(24.06)	21.63(19.98)	18.86(15.23)	14.27(11.21)	<0.001
Mean Fentanyl (mcg/Kg), (SD)	28.38(24.06)	21.63(19.98)	18.86(15.23)	14.27(11.21)	<0.001
Mean Dexmedetomidine (mcg/Kg), (SD)	0.18(1.26)	0.14(0.93)	0.32(1.64)	0.55(2.32)	<0.001
	**Day 3**				
	**n = 116**	**n = 103**	**n = 115**	**n = 67**	
Mean Propofol (mg/Kg), (SD)	20.17(25.30)	17.24(25.36)	11.92(17.65)	7.97(14.91)	<0.001
Mean Fentanyl (mcg/Kg), (SD)	23.10(23.00)	19.96(21.69)	17.02(16.32)	12.29(11.46)	<0.001
Mean Dexmedetomidine (mcg/Kg), (SD)	1.15(4.29)	0.48(1.91)	0.49(1.82)	1.01(4.19)	<0.001

Abbreviations SD standard deviation.

**Fig 2:**
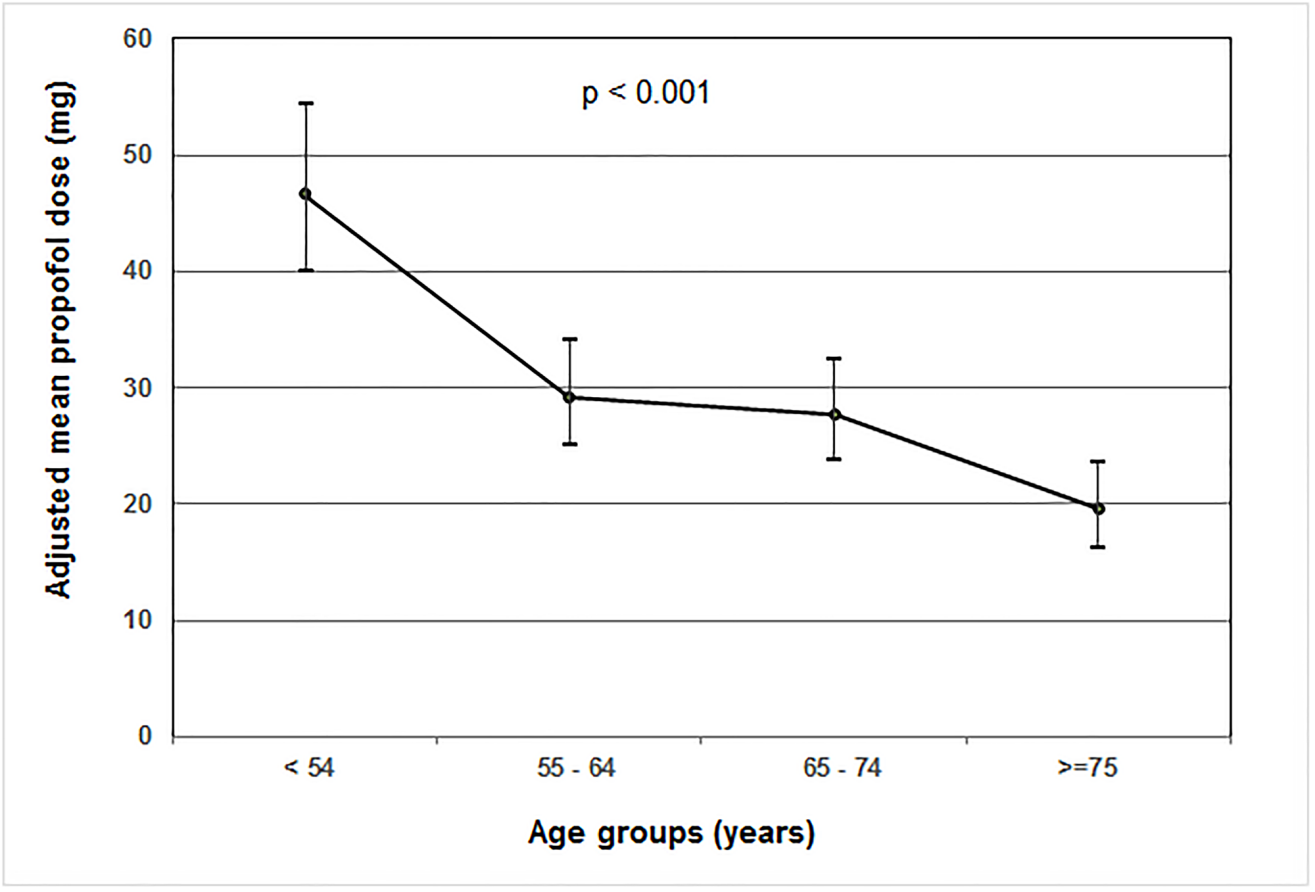
Adjusted mean total dose of propofol according to age groups.

**Fig 3:**
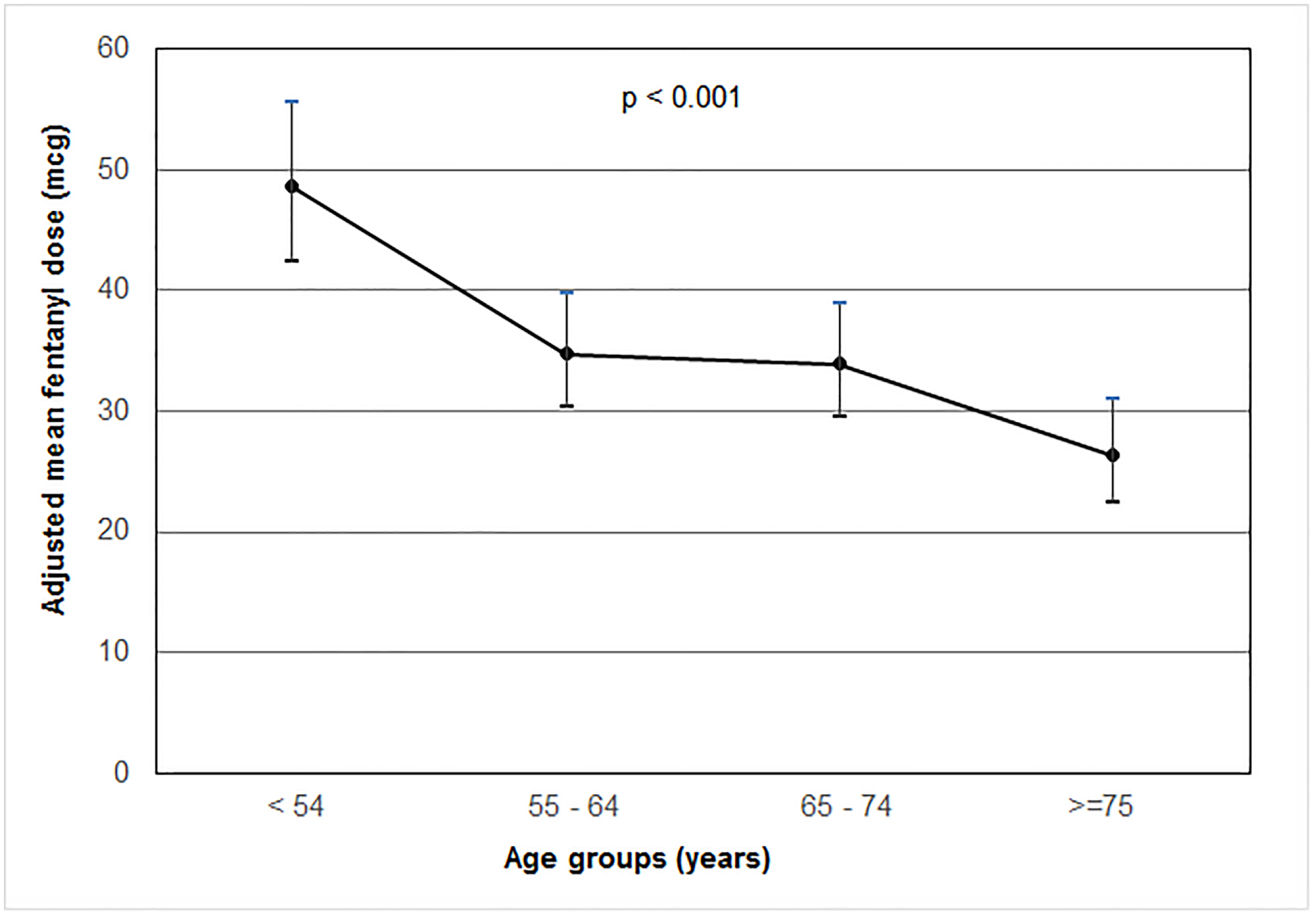
Adjusted mean total dose of fentanyl according to age groups.

Tables 3 and 4 present the univariate and multivariate associations between specific risk factors and total dose of propofol and fentanyl adjusted to weight over the first 72 hours of MV respectively. The univariate analysis showed elderly patients received significantly lower doses of propofol and fentanyl. It also showed that higher doses were significantly associated with vasopressor use, presence of CVL, arterial line and RASS score. Significance of race was more pertinent in the “other” group. After adjustment for gender, diagnosis, CVL, and vasopressor use, increasing age group remains significantly associated with a lower total dose/Kg of both propofol and fentanyl in the multivariate analysis. Figs 2 and 3 present mean doses/Kg of propofol and fentanyl according to age groups, respectively.

**Table 3:** Univariate association between total propofol and fentanyl dose adjusted to weight and individual risk factors.

Risk factors	Total propofol dose			Total fentanyl dose		
	RMD	95% CI	p-value	RMD	95% CI	p
**Gender** (Male v Female)	1.38	1.16–1.66	< 0.001	0.81	0.69–0.95	0.008
**Race**			0.009			0.037
Chinese	Ref			Ref	-	-
Malay	0.948	0.77–1.17	0.624	0.97	0.81–1.16	0.726
Indian	1.21	0.90–1.63	0.202	1.15	0.89–1.49	0.270
Other	1.66	1.20–2.30	0.002	1.46	1.10–1.93	0.008
**Age** (years)			< 0.001			< 0.001
≤ 54	Ref			Ref		
55–64	0.66	0.53–0.83	< 0.001	0.75	0.61–0.91	0.004
65–74	0.66	0.53–0.83	< 0.001	0.77	0.63–0.94	0.010
≥ 75	0.44	0.34–0.57	< 0.001	0.57	0.46–0.71	< 0.001
**APACHE II**	1.00	0.99–1.01	0.911	1.00	0.99–1.01	0.793
**Diagnosis**			0.061			0.003
Sepsis	Ref			Ref		
Airway disease	1.27	0.92–1.77	0.146	1.14	0.86–1.50	0.373
Renal disease	0.94	0.55–1.59	0.804	0.79	0.50–1.24	0.305
CVS disease	0.78	0.51–1.19	0.248	0.73	0.51–1.04	0.083
Neurological disease	0.60	0.24–1.48	0.270	0.41	0.19–0.90	0.025
Malignancy	1.07	0.68–1.67	0.777	0.96	0.65–1.41	0.825
Other	0.73	0.58–0.93	0.012	0.71	0.58–0.87	0.001
**Dialysis**	1.05	0.86–1.27	0.658	1.10	0.93–1.31	0.252
**Vasopressor**	1.59	1.31–1.92	< 0.001	1.54	1.31–1.81	< 0.001
**Transport**	0.89	0.66–1.19	0.426	1.06	0.82–1.37	0.632
**Mobilization**	1.15	0.85–1.56	0.359	1.22	0.94–1.58	0.140
**Central Venous Line**	2.18	1.53–3.10	< 0.001	1.83	1.35–2.47	< 0.001
**Arterial line**	1.76	1.13–2.76	0.013	1.47	1.00–2.16	0.050
**NG/OG tube**	1.07	0.61–1.89	0.808	1.53	0.94–2.49	0.085
**RASS score**			0.006			0.023
Calm / drowsy	Ref			Ref		
Restless / combative	1.57	1.19–2.08	0.002	1.40	1.10–1.78	0.007
Sedated /unarousable	1.31	1.05–1.64	0.017	1.22	1.01–1.48	0.040

Abbreviations RMD relative mean difference, APACHE Acute Physiology and Chronic Health Evaluation, NG/OG Naso/Oro-gastric, RASS Richmond Agitation-Sedation Scale.

**Table 4:** Multivariate association between total propofol and total fentanyl dose adjusted to weight and significant risk factors.

Risk factors	Total propofol dose			Total fentanyl dose		
	RMD	95% CI	p-value	RMD	95% CI	p
**Gender** (Male v Female)	1.27	1.06–1.49	0.007	0.87	0.75–1.01	0.077
**Age** (years)						
≤ 54	Ref					
55–64	0.63	0.50–0.78	<0.001	0.71	0.59–0.87	0.001
65–74	0.59	0.48–0.74	< 0.001	0.70	0.57–0.85	< 0.001
≥ 75	0.42	0.33–0.54	< 0.001	0.54	0.44–0.67	< 0.001
**Diagnosis**						
Sepsis	Ref			Ref		
Airway disease	1.35	0.99–1.84	0.056	1.20	0.92–1.57	0.177
Renal disease	1.08	0.66–1.76	0.771	0.88	0.57–1.35	0.547
CVS disease	0.88	0.60–1.31	0.539	0.80	0.57–1.13	0.212
Neurological disease	0.91	0.39–2.12	0.832	0.57	0.27–1.19	0.133
Malignancy	1.22	0.80–1.85	0.360	1.06	0.74–1.53	0.740
Other	0.74	0.59–0.93	0.009	0.72	0.59–0.88	0.002
**Vasopressor**	1.56	1.28–1.90	< 0.001	1.48	1.25–1.76	< 0.001
**Central Venous Line**	1.64	1.15–2.33	0.006	1.41	1.03–1.91	0.030

Abbreviations RMD relative mean difference, RASS Richmond Agitation-Sedation Scale.

Table 5 shows the effects of doses/Kg of propofol and fentanyl on the cessation of sedation/ analgesia. Doses of propofol/Kg and fentanyl/Kg, vasopressor use, arterial line, CVL and deep sedation were significant in the univariate analysis. In the multivariate analysis, only dose of propofol (mg/Kg) and fentanyl (mcg/Kg) and use of restrainer were significant. Each additional 1mg/Kg of propofol used in the period before the cessation of sedation/analgesia reduced the chance of cessation by 2% independent of other risk factors. Effect of additional fentanyl was less: each 1mcg/Kg of additional fentanyl reduced the chance of cessation by 1%. Use of restrainer reduced the chance of stopping sedation/analgesia by 52% (95% CI 30–78%) compared to the patients without any restrainer.

**Table 5:** Factors associated with cessation of sedation and analgesia.

Patient characteristics	Cessation of sedation and analgesia					
	Univariate			Multivariate		
	OR	95% CI	p	OR	95% CI	p
**Age**, years	1.01	1.00–1.02	0.225			
**Propofol**, mg/Kg	0.97	0.96–0.98	< 0.001	0.98	0.97–0.99	< 0.001
**Fentanyl**, mcg/Kg	0.971	0.965–0.977	< 0.001	0.99	0.98–0.997	0.011
**APACHE I**I	0.98	0.96–1.00	0.079			
**Gender** (Female vs Male)	1.25	0.87–1.78	0.224			
**Race**			0.617			
Malay vs Chinese	1.31	0.86–1.99	0.212			
Indian vs Chinese	1.17	0.65–2.10	0.599			
Others vs Chinese	0.97	0.51–1.82	0.913			
**Use of Restrainer**	0.69	0.47–1.00	0.053	0.48	0.30–0.78	0.003
**Vasopressor**	0.51	0.34–0.76	0.001			
**Central Venous Line**	0.44	0.20–0.96	0.039			
**Arterial Line**	0.11	0.03–0.49	0.004			
**Naso/Orogastric Tube**	0.24	0.05–1.07	0.063			
**Deep Sedation**	0.27	0.19–0.39	< 0.001			

Abbreviations OR Odds ratio, APACHE Acute Physiology and Chronic Health Evaluation.

Thirty-nine patients received no sedation, no analgesia or neither. Their characteristics are shown in S1 Table (group designated as Dose 0). Although statistically not significant due to small number of such patients, there is a trend of not requiring sedation/analgesia with increasing age.

## Discussion

In this study, age was inversely related to the dose of propofol required to maintain a targeted RASS in an ICU where a sedation protocol is routinely used (Tables 3 and 4). Propofol pharmacokinetics follow the classic three compartment model [22]: a small central compartment, one rapidly and one slowly equilibrating peripheral compartments. With advancing age, the central compartment is comparatively smaller [23], potentially resulting in higher plasma propofol concentration in the elderly [24]. Context-sensitive half-time, a measure of 50% plasma decrement time, is prolonged in elderly patients making them slow to recover from propofol infusions [17]. In relation to this, our study showed that for elderly patients aged 75 years and above, the total amount of propofol infused was less than half that of those administered to patients below 55 years of age. On the other hand, age has minimal influence on the pharmacokinetics of fentanyl [25]. However, elderly patients are more sensitive to the increased pharmacodynamic effect of fentanyl to brain, thus requiring approximately half the dose [26]. Consistent with this report, our finding demonstrated that the total of amount fentanyl received by patients aged 75 years and above is 0.54 times that of those administered to younger patients below 55 years of age.

Possible interaction between propofol and fentanyl is an important consideration amongst the elderly patients. Fentanyl may reduce the volume of the central compartment and hence the clearance of propofol [27]. The latter is an inhibitor of cytochrome 450 pathways and may increase plasma concentration of opioids including fentanyl [22]. Propofol reduces preload, myocardial contractility and mean arterial pressure via inhibition of sympathetic vasoconstriction [28]. Consistent with these reports, our study has also found that vasopressor use was independently associated with higher doses of both propofol and fentanyl. Most patients who receive sedation/analgesia also require multiple venous accesses explaining the association of presence of CVL with higher doses propofol and fentanyl. Sicker patients require higher sedation/analgesia, hence the association of vasopressor with CVL is also possible; however, we did not find APACHE II score to be significantly associated with total sedation/analgesia doses suggesting venous access to be the likely reason for the association.

Successful cessation of sedation is a precursor for SBT leading to extubation. Several randomized trials have shown the importance of daily cessation of sedation [7, 29]. Lesser is known about the factors affecting sedation cessation. In this context, deep sedation was associated with longer time to extubation, ICU LOS [7, 29] and mortality [30, 31]. A recent study has also suggested that administering higher dose of sedatives during the previous night was associated with failure to meet SBT criteria the following day [32]. Similarly, we found that increasing the dose of propofol/Kg during the period before cessation was associated with reduced chance of cessation independent of the sedation level (Table 5). Physical restrainers are used in ICU to prevent unintended treatment interruptions like self-extubation. They often present an ethical dilemma, are viewed as a restriction to patients’ autonomy and may even worsen delirium [33–35]. In our study, the use of restrainers was associated with a reduced chance of stopping sedation. Restrainer use may be more common in agitated patients and as such, bedside nurses may feel less confident about stopping sedation without any risk of adverse events.

How can the current data be useful to a pragmatic ICU team? Age related changes in the pharmacokinetics and pharmacodynamics properties of sedatives and analgesics need to be considered separately. It is not adequate to monitor patients based on clinical scores alone, as is currently done. This will involve reducing doses in the elderly patients, achieving a RASS score close to 0 and expecting slower recovery from sedation which may not warrant additional investigations. Hypovolemic patients have reduced central compartment [36] and it may be necessary to avoid bolus doses of propofol [37], particularly in the elderly who already have smaller central compartment. Keeping the doses of propofol and fentanyl to the minimum will facilitate the cessation of sedation. The association between the restrainer use and reduced cessation of sedation is intriguing but will need further validation. With a world-wide trend of aging population, more than half of the current patient population in ICU is above 65 years of age and this is likely to increase in the future [38]. Understanding the interaction between commonly used sedatives/analgesics and their effect on elderly patients with multiple comorbidities is important for a vigilant ICU team and we expect that the future guidelines will include specific recommendations. Since the elderly patients received less sedation/analgesia without having specific dose ranges for different age groups, current study emphasizes the importance of a protocol driven sedation. However, it is to be noted that the RASS is an ordinal scale and a unit increment or decrement in the score does not mean a patient has moved equal distance up or down the scale. Finer adjustments guided by patient related factors may allow better management of sedation leading to improved outcomes.

We acknowledge the several weaknesses in this study. First, this was a single center observational study and although we attempted to account for all variables that may contribute to the outcome, residual confounding by the unmeasured variables is possible. Second, we have considered only two commonly used sedatives and analgesics in our ICU. Although they are frequently used drugs [39] and recommended by the PAD guidelines [5], other institutions may use different medications and hence our findings may not be directly applicable. Third, we did not include data regarding the occurrence of delirium in ICU due to incomplete documentation by the nurses. Besides, their visual assessment of pain and documentation of pain score was inconsistent; therefore, we could not include this information in the analysis.

## Conclusion

Our present study suggests that patient related factors such as age, gender, ICU procedures (e.g. presence of CVL) and drugs used (e.g. vasopressor) affect the doses of sedatives and analgesics. Reducing dose of sedative/analgesic irrespective of depth of sedation and judicious use of restrainers are modifiable factors that may lead to early cessation of sedation. In addition to using a clinical protocol, further refinement of dose may be possible based on the patient related factors with improved outcomes.

## Supporting information

S1 Table Demographic and clinical characteristics of study subjects with no sedation/no analgesia or no sedation and no analgesia (Dose 0) and both sedation and analgesia (Dose > 0). Abbreviations APACHE Acute Physiology and Chronic Health Evaluation, CVL Central venous line, NG nasogastric.
(DOCX)
