# Supplementary material for: Age related inverse dose relation of sedatives and analgesics in the intensive care unit
Source: PLoS One. 2017 Sep 28;12(9):e0185212. doi: 10.1371/journal.pone.0185212 (PMC5619735; doi:10.1371/journal.pone.0185212)
Supplement: S1 Table — Abbreviations APACHE Acute Physiology and Chronic Health Evaluation, CVL Central venous line, NG nasogastric. (DOCX) [file pone.0185212.s001.docx]

**S1 Table**: Demographic and clinical characteristics of study subjects with no sedation/no analgesia or no sedation and no analgesia (Dose 0) and both sedation and analgesia (Dose > 0)

| **Characteristics** | Total  (n = 576) | Dose 0  (n = 39) | Dose > 0  (n = 537) | p |
| --- | --- | --- | --- | --- |
| **Gender** (%) |  |  |  | 0.041 |
| Male | 368 (63.9) | 19 (48.7) | 349 (65.0) |  |
| Female | 208 (36.1) | 20 (51.3) | 188 (35.0) |  |
| **Race** |  |  |  | 0.715 |
| Chinese | 338 (58.7) | 20 (51.3) | 318 (59.2) |  |
| Malay | 135 (23.4) | 12 (30.8) | 123 (22.9) |  |
| Indian | 57 (9.9) | 4 (10.3) | 53 (9.9) |  |
| Other | 46 (8.0) | 3 (7.7) | 43 (8.0) |  |
| **Age** (years) |  |  |  | 0.080 |
| ≤ 54 | 157 (27.3) | 8 (20.5) | 149 (27.8) |  |
| 55 – 64 | 153 (26.6) | 8 (20.5) | 145 (27.0) |  |
| 65 – 74 | 151 (26.2) | 9 (23.1) | 142 (26.4) |  |
| ≥ 75 | 115 (20.0) | 14 (35.9) | 101 (18.8) |  |
| **Mean weight, Kg (SD)** | 63.4 (18.2) | 55.3 (14.9) | 64.0 (18.3) | 0.004 |
| **Mean APACHE II (SD)** | 28.2 (8.1) | 31.9 (8.0) | 27.9 (8.0) | 0.003 |
| **Diagnosis** |  |  |  | 0.007 |
| Sepsis | 364 (63.2) | 21 (53.9) | 343 (63.9) |  |
| Airway disease | 43 (7.5) | 1 (2.6) | 42 (7.8) |  |
| Renal disease | 19 (3.3) | 4 (10.3) | 15 (2.8) |  |
| CVS disease | 24 (4.2) | 0 (0.0) | 24 (4.5) |  |
| Neurological disease | 5 (0.9) | 0 (0.0) | 5 (0.9) |  |
| Malignancy | 21 (3.7) | 0 (0.0) | 21 (3.9) |  |
| Other | 100 (17.4) | 13 (33.3) | 87 (16.2) |  |
| **Dialysis** | 154 (26.7) | 13 (33.3) | 141 (26.3) | 0.335 |
| **Vasopressor** | 415 (72.1) | 13 (33.3) | 148 (27.6) | 0.438 |
| **Transport** | 55 (9.6) | 4 (10.3) | 51 (9.5) | 0.876 |
| **Mobilization** | 51 (8.9) | 3 (7.7) | 48 (8.9) | 0.791 |
| **CVL** | 537 (93.2) | 34 (87.2) | 503 (93.7) | 0.119 |
| **Arterial line** | 552 (95.8) | 36 (92.3) | 516 (96.1) | 0.254 |
| **NG tube** | 562 (97.6) | 38 (97.4) | 524 (97.6) | 0.955 |
| **RASS score** |  |  |  | 0.008 |
| Calm / drowsy | 120 (20.9) | 14 (35.9) | 106 (19.8) |  |
| Restless / combative | 96 (16.7) | 1 (2.6) | 95 (17.7) |  |
| Sedated / unarousable | 359 (62.4) | 24 (61.5) | 335 (62.5) |  |

Abbreviations APACHE Acute Physiology and Chronic Health Evaluation, CVL Central venous line, NG nasogastric
